# Effect of additives on the synthesis efficiency of nanoparticles by laser-induced reduction

**DOI:** 10.3762/bjnano.16.35

**Published:** 2025-03-27

**Authors:** Rikuto Kuroda, Takahiro Nakamura, Hideki Ina, Shuhei Shibata

**Affiliations:** 1 illuminus Inc., 307 Wako Riken Incubation Plaza, 2–3–13, Minami, Wako, Saitama, 351–0104, Japan

**Keywords:** laser-induced reduction, laser synthesis and processing of colloidal nanoparticles, production efficiency

## Abstract

Laser-induced reduction in liquid (LRL) is a physicochemical technique for synthesizing nanoparticles by irradiating a solution containing metal ions with a high-intensity laser. It is simple and environmentally friendly, as it does not require reducing agents or high-temperature, high-pressure environments. In this method, nanoparticles are synthesized by reducing metal ions with short-lived radical species produced by the breakdown of solvent molecules in a high-intensity reaction field near the focus of the laser. This unique reaction has the characteristic of being able to synthesize non-equilibrium solid–solution alloy nanoparticles. On the other hand, it is necessary to improve the synthesis efficiency of nanoparticles in large quantities for practical use. In this study, we investigated improvements of the synthesis efficiency of nanoparticles in LRL by adding scavengers, such as isopropyl alcohol (IPA) and glycerin, for oxidative radicals formed by laser irradiation to the solution and converting the oxidative radicals into reducing species. Based on the evaluation of the synthesis efficiency of Au nanoparticles, it was confirmed that the addition of IPA increased the synthesis efficiency of nanoparticles by about five times, and the addition of glycerin increased it by about nine times. Furthermore, by adding these oxidizing radical scavengers, it became possible to synthesize nanoparticles even when the concentration of metal ions in the solution was increased. And as a result, the synthesis efficiency of nanoparticles increased by more than 18 times. This means that it is possible to synthesize 160 mg/h of Au nanoparticles in the current system. It was also shown that non-equilibrium solid–solution alloy nanoparticles could be synthesized even when a radical scavenger was added. Furthermore, the addition of a radical scavenger also made it possible to synthesize base metal nanoparticles, which have been difficult to synthesize using the LRL. In addition, the efficiency of nanoparticle synthesis has been dramatically improved, and the variety of materials that can be produced has increased. This expands the potential of nanoparticles synthesized by LRL to be used in industrial applications.

## Introduction

Metal nanoparticles exhibit unique chemical, physical and optical properties that are not found in their bulk materials, and are used in a variety of fields including electrode materials [1], conductive pastes [2–3], catalysts [4–5], sensors [6–8], and drug delivery systems [9]. The chemical reduction [10] and the solvothermal methods [11–12] are well known for synthesizing nanoparticles in large quantities at low cost, but these methods require the use of chemical substances such as reducing agents and stabilizers that should be purified and removed after synthesis. In some cases, control of temperature and/or pressure are also required during reaction. In contrast, laser processing in liquids, which does not require chemical substances as reducing agents and can form nano- and submicron particles at room temperature and atmospheric pressure, has attracted much attention as a simple and environmentally friendly particle synthesis technique. Laser-based particle synthesis methods can be roughly divided into two categories depending on whether the target source material is a ‘solid’ or a ‘metal ion’. Methods for synthesizing particles using solid materials include laser ablation in liquid (LAL) [13–15], laser fragmentation in liquid (LFL) [16], and laser melting in liquid (LML) [17], and many excellent reports have been published on the synthesis of various nanoparticles that maintain the crystal structure and composition of the source solid material.

In contrast to those methods, laser-induced reduction in liquid (LRL) is a nanoparticle synthesis method based on reduction reactions induced by laser in solution. Synthesis of nanoparticles of various materials [18–24] using different mechanisms [25–28] have been conducted via LRL. High-energy ultrashort pulses focused and irradiated into a solution cause the breakdown of solvent molecules. Then, produced solvated electrons among reactive species reduce the metal ions in the solution to form nanoparticles [25–26]. It has been reported that, due to the short reduction reaction of solvated electrons [29–30], it is possible to form solid–solution alloy nanoparticles with controlled compositions that do not exist in the equilibrium phase diagram [20]. It has been reported that alloy nanoparticles exhibit superior properties to conventional pure metal nanoparticles. For example, CoNiCuZnPd alloy nanoparticles supported on CeO_2_ show higher catalytic properties for the decomposition of NO than that of Pd nanoparticles [31]. MoZnFeCoNi is also more active as a water electrolysis catalyst than IrO_2_ [32]. The combination and composition of elements are important to achieve superior properties to those of pure metal nanoparticles. The typical method for nanoparticles synthesis such as chemical reduction with thermal equilibrium reaction is basically limited to the formation of alloy nanoparticles in an equilibrium state. On the other hand, since LRL can form non-equilibrium alloy nanoparticles, it has the potential to provide alloy nanoparticles with properties that have not been yet possible to be obtained.

The synthesis efficiency of the current system using LRL has been improved from 1 mg/h [18], when this phenomenon was first discovered, to 20 mg/h through improvements to the optical system and scale-up. However, further improvements in the synthesis efficiency of nanoparticles are an urgent issue to put the nanoparticles synthesized by LRL to practical use.

In this study, we attempted to improve the synthesis efficiency of nanoparticles using LRL by controlling the reaction environment of the solution during laser irradiation.

## Results and Discussion

### Increasing the synthesis efficiency of nanoparticles via laser-induced reduction by adding radical scavengers

In laser-induced reduction, laser irradiation breaks down water molecules to produce various radical species. The standard electrode potentials of solvated electrons (*e*^−^_aq_) and hydrogen radicals (H^•^) are *E*^0^ = −2.77 V and *E*^0^ = −2.1 V, respectively [33]. It has been reported that solvated electrons reduce metal ions and contribute to the formation of nanoparticles [25–26].


[1] 2H2O→e aq−+H3O++O⋅H

[2] H2O→H⋅+O⋅H

On the other hand, the standard electrode potential of the hydroxyl radical (^•^OH) is +2.7 V, and it is a strong oxidizing species [33], so it is thought that it oxidizes the reduced metal atoms and clusters, inhibiting nanoparticle synthesis. It was assumed that the synthesis of nanoparticles would be promoted by removing the hydroxyl radicals formed by laser irradiation. Isopropyl alcohol acts as a radical scavenger, and reacts with ^•^OH to produce a reducing radical (*E*^0^ = −1.8 V) [34].


[3] CH3CH2(OH)CH3+O⋅H→CH3C⋅H(OH)CH3+H2O

[4] $${\text{CH}}_{3}{\text{C}}^{\cdot }\text{H}(\text{OH}){\text{CH}}_{3}+{\text{H}}_{2}\text{O}\to {\text{CH}}_{3}{\text{COCH}}_{3}+e^{-}{\text{+H}}_{3}{\text{O}}^{+}$$

Therefore, we added IPA to the solution and compared the results with the aim of improving the synthesis efficiency of nanoparticle by LRL.

**Figure 1:**
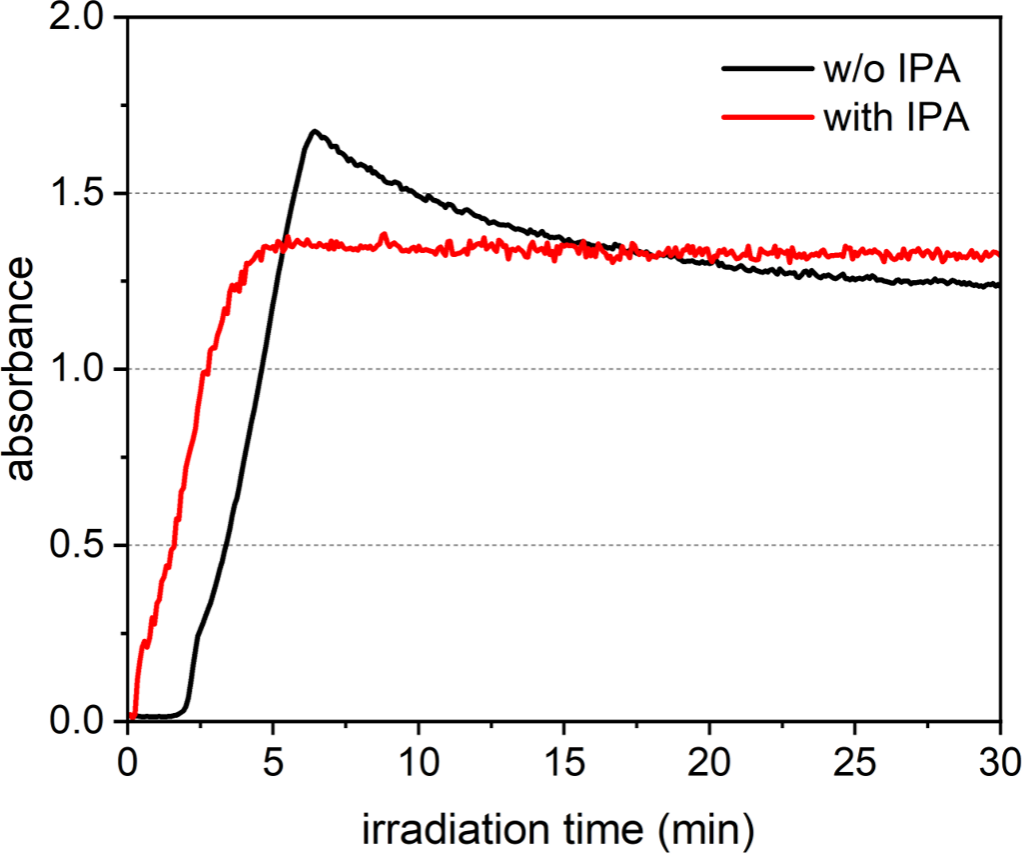
Time variation in absorbance at a wavelength of 520 nm in the UV–vis absorption spectrum of the solution during laser irradiation without (black line) and with (red line) IPA.

**Figure 2:**
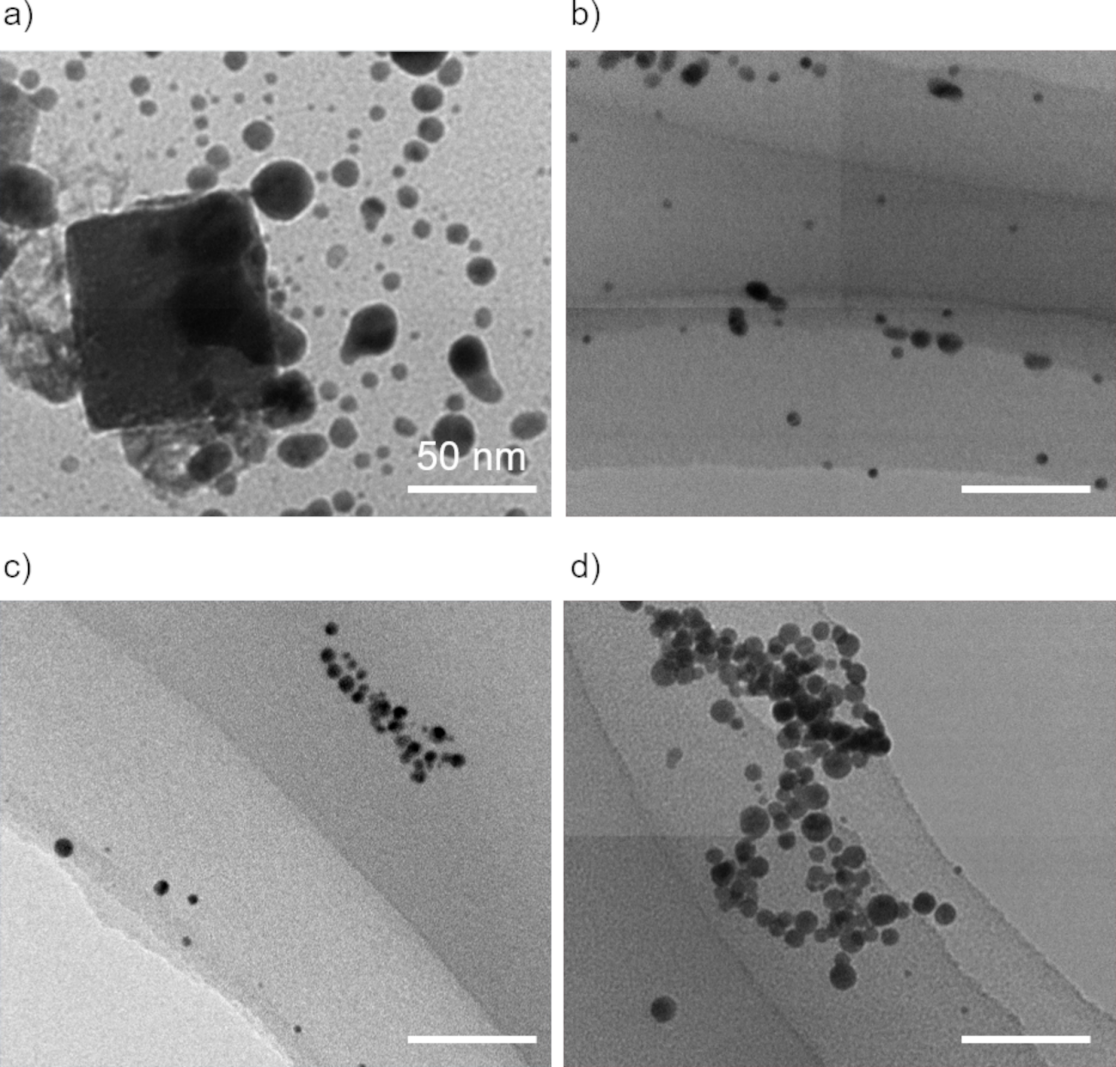
TEM images of Au nanoparticles synthesized by laser irradiation into a tetrachloroauric acid solution a, b) without and c, d) with IPA. These samples were taken from the solution a, c) 10 min and b, d) 30 min after laser irradiation.

The UV–visible absorption spectrum of the aqueous solution of gold chloride was measured every 5 s during laser irradiation to evaluate the progress of the reaction. Figure 1 shows the results of the change in the absorption peak at 520 nm in the UV–vis absorption spectrum caused by the localized surface plasmon resonance (LSPR) of the Au nanoparticles as a function of the laser irradiation time. The black line shows the change in absorbance for the solution without IPA, and the red line shows those for the solution with 10 vol % IPA. In both cases, the absorbance increases as the laser irradiation time progresses, reaches a peak, and then becomes constant. In the case of the absorbance spectrum of the solution without IPA (Figure 1, black line), the absorbance gradually increases from about 2 min after laser irradiation, reaches a maximum after 6 min, and then decreases to a constant value at 26 min. This is assumed to be based on the following mechanism. 1) From the start of laser irradiation to 2 min: equilibrium between nucleation due to ion reduction and atom re-dissolution due to oxidation; 2) 2–6 min of laser irradiation: particle formation and crystal growth reaction; 3) 6–26 min of laser irradiation: continuous reduction and particle size reduction by laser fragmentation in liquid. Therefore, this means that all reactions are complete after 26 min of laser irradiation in the solution without IPA. On the other hand, when 10 vol % IPA was added (red line in Figure 1), an increase in absorbance was immediately seen after the start of laser irradiation, and the absorbance levelled off after 5 min of laser irradiation. Figure 2 shows the transmission electron microscopy (TEM) images of the samples extracted from solutions with and without and IPA after 10 and 30 min of laser irradiation. In the case of the sample without IPA, in the TEM image of the sample after 10 min of laser irradiation, which is the initial stage of laser fragmentation, in addition to spherical particles with a wide particle size distribution of >10 nm, particles with a square shape of >50 nm were also observed. It is thought that the square-shaped particles were formed by crystal growth of the atoms produced by laser irradiation as nuclei, while consuming unreacted ions in the solution through a self-catalytic effect. This also suggests that the reduction reaction was not complete after 10 min of laser irradiation. The sample after 30 min of laser irradiation shows that particles with a narrow particle size distribution of less than 10 nm in diameter were formed. In contrast, in the sample with 10 vol % IPA, even in the TEM image of the sample after 10 min of laser irradiation, nanoparticles with a narrow particle size distribution of less than 10 nm in diameter were observed. This suggests that the nanoparticle synthesis reaction finished after 10 min of irradiation. This is due to the fact that the addition of IPA converted the hydroxyl radicals produced by laser irradiation into reducing species, and the reduction reaction proceeded efficiently. Furthermore, this reaction is particularly pronounced in the initial stage of nucleation. In the case without IPA, there was no increase in absorbance due to the formation of nanoparticles until about 2 min after laser irradiation, whereas in the case with IPA, the absorbance immediately increased after laser irradiation. This suggests that IPA functions as a radical scavenger. From these results, it was clear that the addition of IPA increased the efficiency of Au nanoparticle synthesis using LRL by about five times. By applying these results to the current synthesis system (20 mg/h), the synthesis efficiency can reach 100 mg/h.

**Figure 3:**
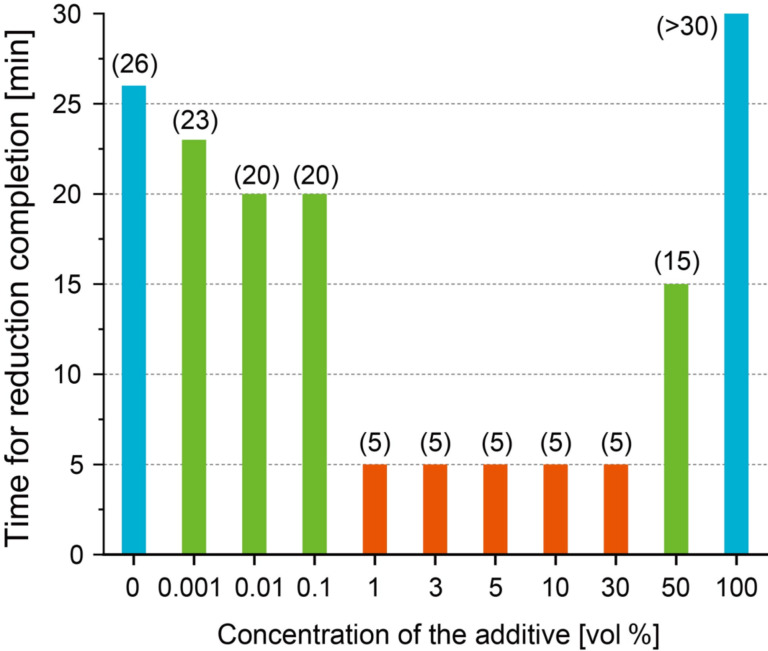
The reaction finishing times of the nanoparticle synthesis by LRL with various IPA concentrations determined from the change in absorbance at a wavelength of 520 nm in the UV–vis absorption spectrum.

We also investigated the optimal concentration of IPA as a radical scavenger. Figure 3 shows the reaction finishing time of the nanoparticles, as determined from the change in absorbance at a wavelength of 520 nm in the UV–vis absorption spectrum of solutions with different concentrations of IPA during laser irradiation. The numbers in parentheses in the figure indicate the reaction finishing time of the nanoparticle synthesis. From these results, it was found that the efficiency of nanoparticle synthesis was improved even under conditions with 0.001 vol % IPA (23 min). In addition, it was also shown that the reaction finishing time was the shortest at 5 min when the IPA concentration was between 1 vol % and 30 vol %. We can conclude that these were suitable IPA concentrations for improving the synthesis efficiency of nanoparticles in LRL. On the other hand, when the IPA concentration was 50 vol %, the reaction finishing time was long (15 min), and when the IPA concentration was 100 vol %, the reaction did not complete even after 30 min of laser irradiation, which was longer than the case without IPA. This is thought to be based on the fact that the relative permittivity of IPA (20.18) is lower than that of water (80.1) [35]. Then the efficiency of radical generation accompanying the decomposition of the solvent by laser irradiation is low.

**Figure 4:**
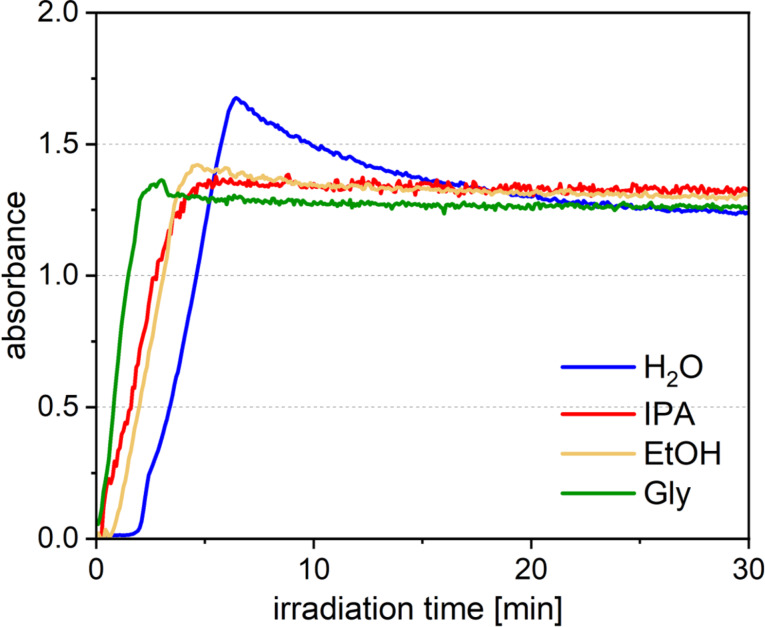
Time variation of absorbance of the solution during laser irradiation at a 520 nm wavelength without scavengers (blue) and with IPA (red), ethanol (yellow), and glycerin (green).

We also investigated the effect of other additives other than IPA as radical scavengers. Figure 4 shows the change in absorbance at a wavelength of 520 nm in UV–vis absorption spectra of an Au solution with IPA, ethanol, and glycerin during laser irradiation. In all cases, the reaction finishing time was shorter than that of the solution without additives. When ethanol was used, the reaction finishing time was almost the same in the case of IPA, but when glycerin was used, the nanoparticle synthesis efficiency was improved by 1.6 times. Therefore, compared to the case without additives, the efficiency of nanoparticle synthesis increased by a factor of about 9 (Au nanoparticle synthesis efficiency in the current system: 160 mg/h) when glycerin was added. We think that the radicals produced by reacting with hydroxyl radicals are stabilized by hyperconjugation with the two methyl groups in IPA, so the stability of the radicals is higher than in ethanol, which has only one methyl group. However, there was no significant difference in the synthesis efficiency when using these additives. So, the stability of the radical has little effect on the synthesis efficiency of Au nanoparticles by LRL. On the other hand, the synthesis efficiency is even higher with glycerin. While IPA and ethanol are monovalent alcohols with a single hydroxy group, glycerin is a trivalent alcohol with three hydroxy groups. Therefore, we speculate that glycerin was able to more efficiently trap oxidizing species such as hydroxyl radicals since it has more reaction points than monovalent alcohols. When considering industrial applications, optimization using additives is essential not only in terms of the efficiency of nanoparticle synthesis but also cost, by-products, and solvent compatibility. We will conduct additional experiments to clarify the specific mechanism of reaction and attempt to select the optimal radical scavenger.

### Increasing the efficiency of nanoparticle synthesis by changing solution concentration with radical scavengers

**Figure 5:**
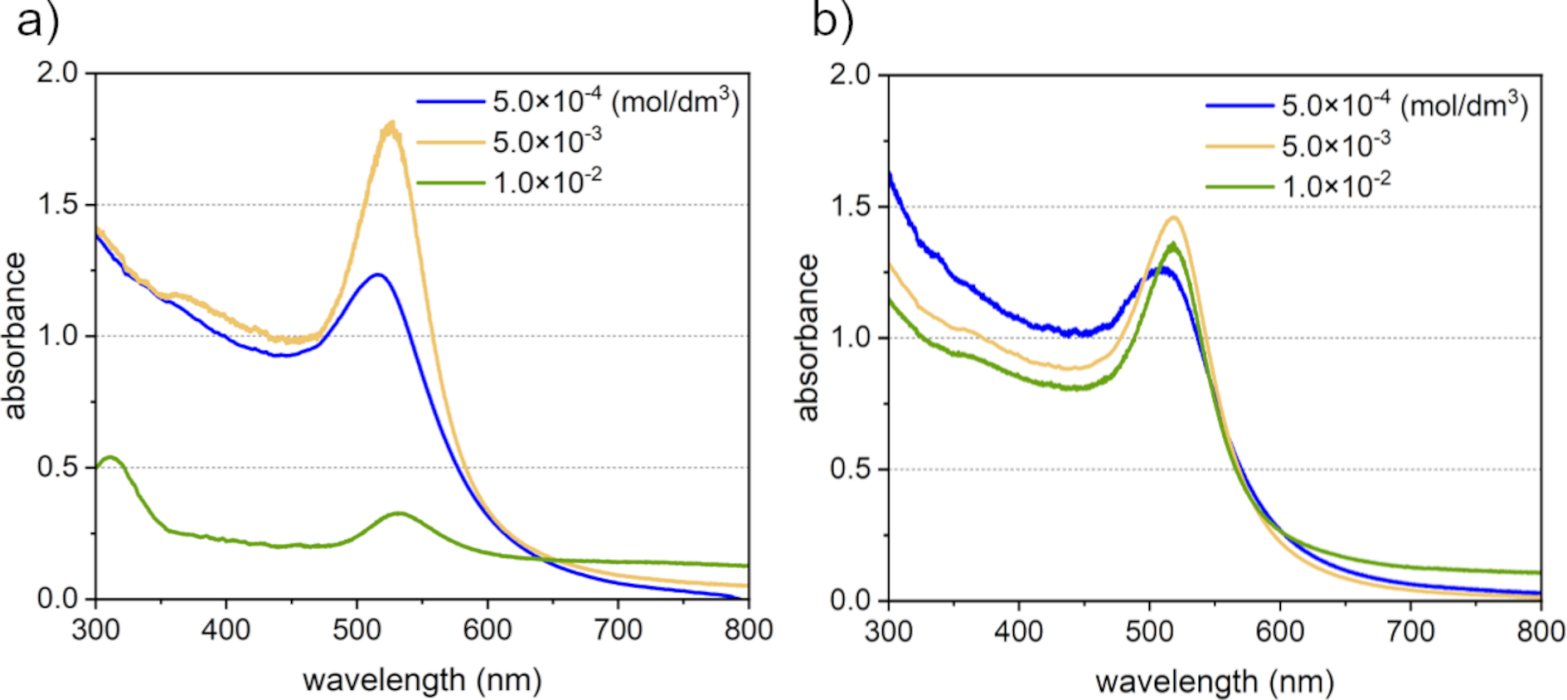
UV–vis absorption spectrum after 30 min of laser irradiation in aqueous solutions of tetra chloroauric acid with various concentrations. a) Without glycerin, b) with glycerin.

When the precursor ion concentration is increased in the synthesis of gold nanoparticles using LRL, the reduction reaction caused by laser irradiation does not sufficiently occur under the same laser irradiation conditions. The crystal growth progresses through equilibrium reactions with unreacted ions due to the self-catalytic effect of the generated nanoparticles [25] and the particles become larger. On the other hand, since the synthesis efficiency was improved by adding scavengers, it was predicted that the synthesis of nanoparticles would be promoted even if the concentration of the ion in solution was increased. The ion concentration was changed from 5.0 × 10^−4^ to 1.0 × 10^−2^ mol/dm^3^, and laser was irradiated for 30 min. Figure 5 shows the absorption spectra of Au ion solutions with different concentrations a) without and b) with glycerin after laser irradiation. In this case, given that the absorbance of the solutions at concentrations of 5.0 × 10^−3^ and 1.0 × 10^−2^ mol/dm^3^ was too high to properly measure the absorption spectrum, all solutions were diluted with pure water to a concentration of 5.0 × 10^−4^ mol/dm^3^ before measurements. The absorption spectrum of the aqueous solution with a concentration of 5.0 × 10^−3^ mol/dm^3^ (10 times higher than that of the standard conditions) without IPA showed a red shift in the absorption peak position, suggesting coarsening of the particles (Figure 5a, yellow). In addition, at a solution concentration of 1.0 × 10^−2^ mol/dm^3^ (20 times higher than that of the standard conditions), an absorption peak at a wavelength of 300 nm derived from the gold chloride ion was observed (Figure 5a, green). This suggested that the nanoparticle synthesis reaction was not complete even after 30 min of laser irradiation. On the other hand, when 10 vol % glycerin was added, the absorption peak wavelengths of the solutions with concentrations of 5.0 × 10^−3^ and 1.0 × 10^−2^ mol/dm^3^ (Figure 5b, yellow and green) were almost the same as that of the solution with a concentration of 5.0 × 10^−4^ mol/dm^3^ (Figure 5b, blue). This indicates that gold nanoparticles with similar optical properties were formed in both cases. However, in the solution with a concentration of 1.0 × 10^−2^ mol/dm^3^, an increase in absorbance was observed in the long wavelength region of the absorption spectrum. Therefore, we evaluated the dispersion state of nanoparticles using dynamic light scattering (DLS) measurements. The obtained particle size distribution showed three peaks at 3.4 nm, 25.5 nm, and 154.0 nm with the volume ratios of 99.34 vol %, 0.64 vol %, and 0.02 vol %, respectively. This suggests that the volume ratio of the nanoparticle aggregates is 0.66 vol %. Therefore, it may be necessary to adjust the dispersion state by adding a dispersant depending on the application. We think that the effect on overall characteristics will be minimal in practice use.

In this experiment, nanoparticle synthesis was already completed after 30 min of laser irradiation even when the concentration of the solution was increased by 20 times. The reaction finishing time for nanoparticles synthesis using a solution with a standard concentration (concentration 5.0 × 10^−4^ mol/dm^3^) is approximately 3 min, and longer synthesis times are required when the solution concentration is increased. However, even when using a solution 20 times more concentrated than the standard concentration, nanoparticle synthesis was confirmed after 30 min of laser irradiation. Therefore, we think that the synthesis efficiency had improved by at least two times. Accordingly, we estimate that the synthesis efficiency in the current system was improved to at least 320 mg/h.

### Synthesis of solid–solution alloy nanoparticles by LRL with radical scavengers

**Figure 6:**
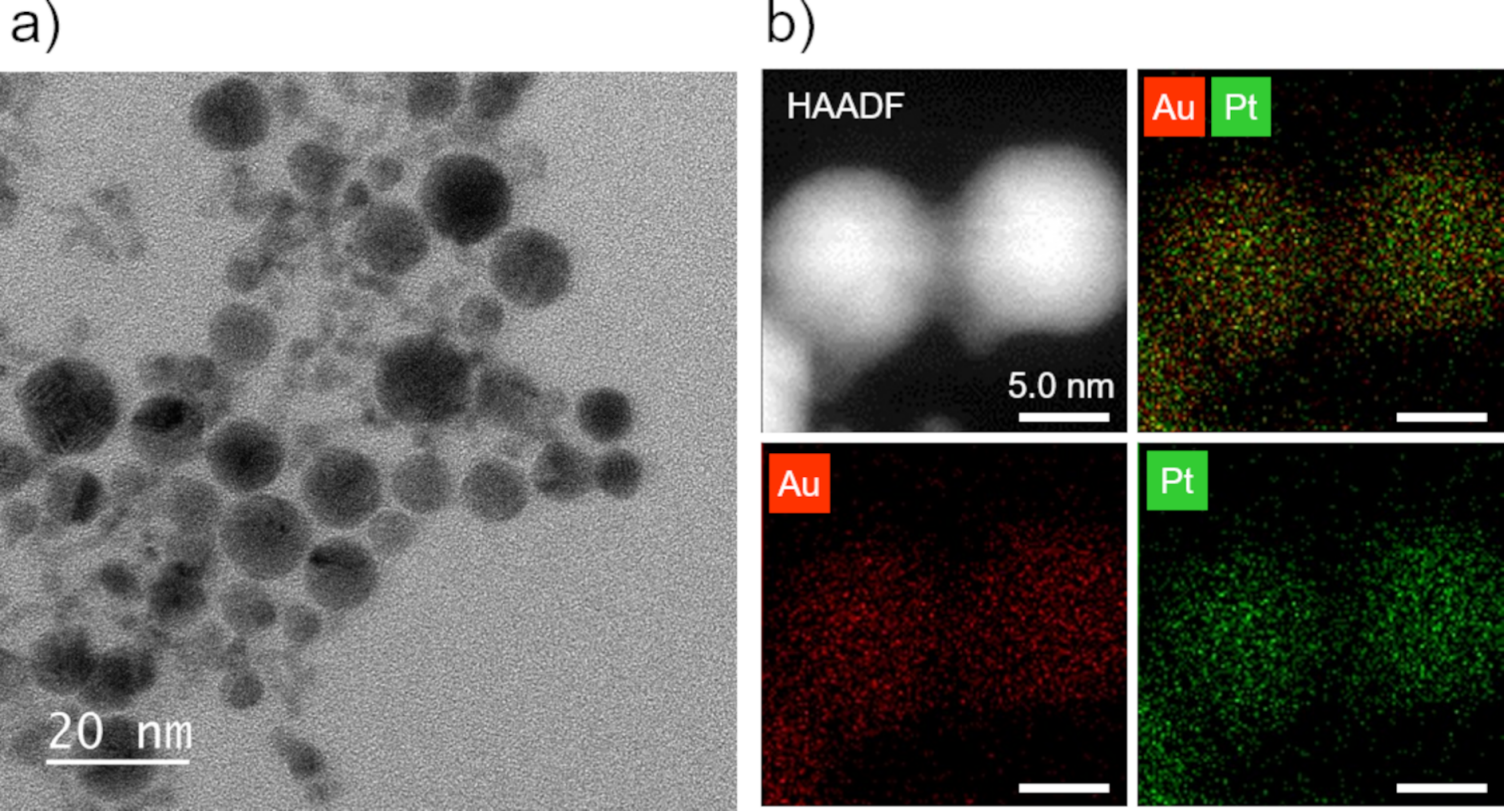
a) A TEM image, b) a high-angle annular dark-field (HAADF) image and STEM-EDS mappings of Au–Pt alloy nanoparticles (atomic ratio Au/Pt = 1:1) synthesized by LAL with IPA as a scavenger.

LRL is a non-equilibrium nanoparticle synthesis technique, and its main feature is that it can form solid–solution alloy nanoparticles of metals that do not dissolve in the bulk form [19–20]. On the other hand, the addition of radical scavengers for promoting the reaction may generate relatively long-lived radicals that are more stable than solvated electrons formed by laser irradiation (Equation 4), and make it difficult to form solid–solution alloy nanoparticles through non-equilibrium reactions. Therefore, we attempted to synthesize alloy nanoparticles by LRL with the addition of IPA as a radical scavenger to a solution containing multiple metal ions, and the structure of the synthesized nanoparticles was evaluated by scanning transmission electron microscopy-energy dispersive spectroscopy (STEM-EDS). In this case, we selected an Au–Pt alloy (atomic ratio, Au/Pt = 1:1) that has an immiscible gap in the binary phase diagram and is difficult to form a solid–solution alloy in a bulk form. Figure 6 shows a a) TEM image and b) STEM-EDS mappings of the particles produced after laser irradiation. The TEM results (Figure 6a) confirmed the formation of nanoparticles with a diameter of less than 20 nm. The STEM-EDS mappings (Figure 6b) clearly showed that Au and Pt were uniformly present within each particle, and the atomic ratio was Au/Pt = 1:1, which was consistent with the mixing ratio of the precursor ions. Namely, it was possible to synthesize non-equilibrium solid–solution alloy nanoparticles by LRL even when a radical scavenger was added to improve the reaction efficiency.

### Synthesis of base metal nanoparticles by LRL with radical scavengers

**Figure 7:**
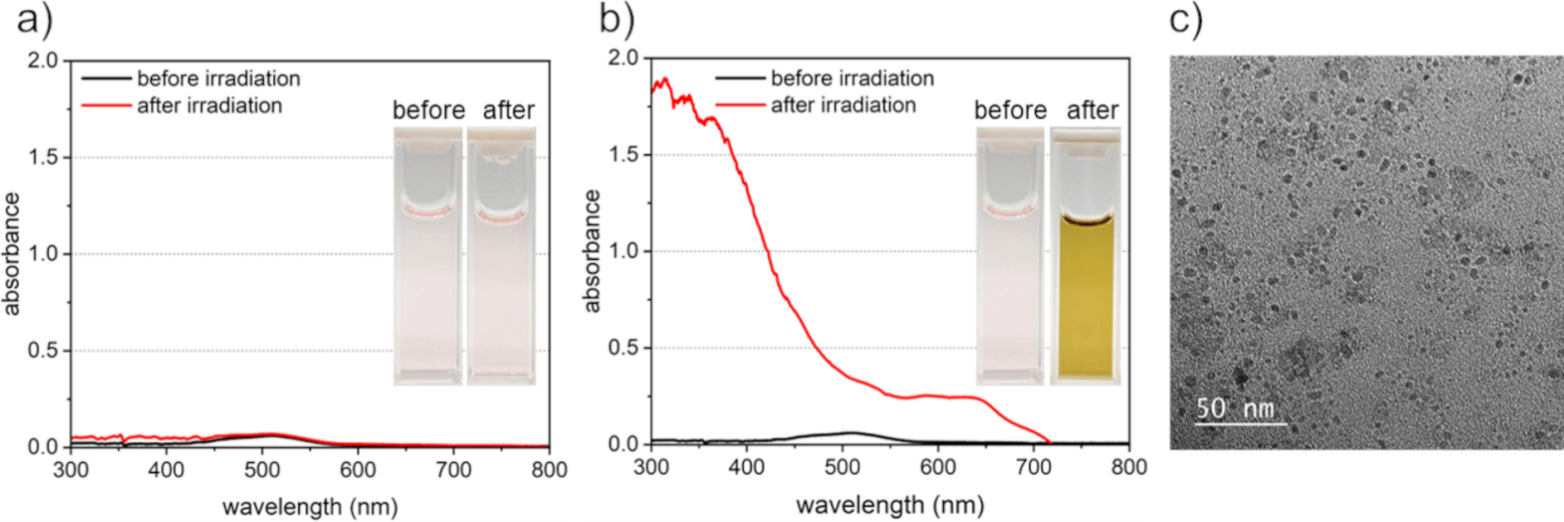
UV–vis absorption spectrum after 60 min of laser irradiation in aqueous solutions of cobalt nitrate a) without and b) with IPA as a scavenger. The inset images are photographs of the appearance of each solution before and after laser irradiation. c) A TEM image of Co nanoparticles synthesized by LAL with IPA. The synthesized nanoparticles might be oxides.

Since the addition of radical scavengers can inhibit the re-dissolution of formed atoms by oxidation, we speculated that it would also be possible to synthesize base metal nanoparticles, which had been difficult by LRL in aqueous solutions. We, therefore, tried to synthesize Co nanoparticles (standard electrode potential: *E*^0^ = −0.28 V [33]). Figure 7a and Figure 7b show the features and spectra of the Co solution before and after laser irradiation with and without 10 vol % IPA as a radical scavenger, and Figure 7c shows a TEM micrograph of the sample after laser irradiation. The feature and absorbance spectrum of the solution did not change before or after laser irradiation, and no nanoparticles were formed without IPA (Figure 7a). On the other hand, when 10 vol % IPA was added as a radical scavenger, the color of the solution changed from pale pink to yellow by laser irradiation (Figure 7b). In the absorbance spectrum after laser irradiation, the absorbance at shorter wavelengths became higher due to Rayleigh scattering by nanoparticles formed in the solution. TEM images of the sample after laser irradiation confirmed the formation of nanoparticles of around <10 nm (Figure 7c).

**Figure 8:**
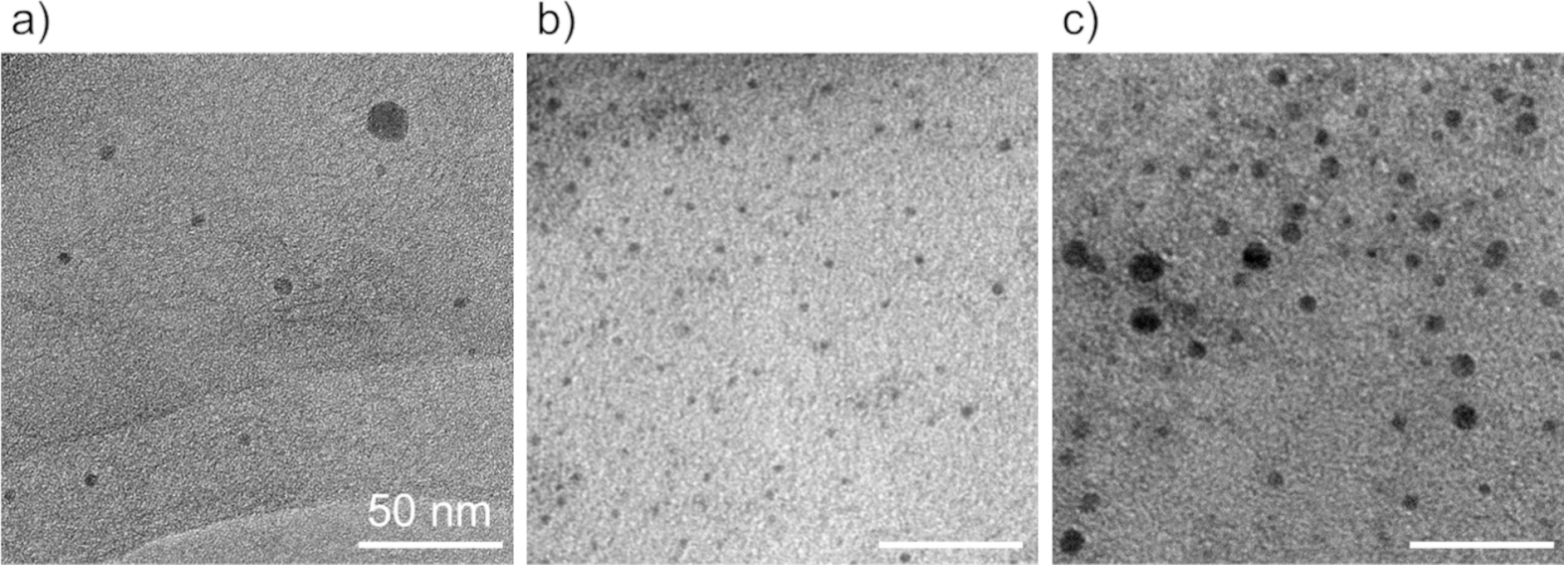
TEM images of a) Hf, b) Al, and c) Y nanoparticles synthesized by LAL with IPA. The synthesized nanoparticles might be oxides.

Based on the above results, we investigated the possibility of using LRL to synthesize base metals. In addition to Co, we also targeted Hf (*E*^0^ = −1.55 V), Al (*E*^0^ = −1.662 V), and Y (*E*^0^ = −2.3 V), which have even more negative reduction potentials than that of Co (*E*^0^ = −0.28 V) [33]. The TEM results for each sample after laser irradiation are shown in Figure 8. The formation of nanoparticles with a diameter of less than 10 nm was confirmed in all samples. Since materials with a more negative reduction potential than Y react with water and dissolve at room temperature, it is difficult to verify the formation of their nanoparticles in water as a solvent. However, in principle, it was shown that materials with a reduction potential lower than that of solvated electrons formed by laser irradiation (*E*^0^ = −2.77 V) could be potentially synthesized by LRL.

## Conclusion

In this study, we aimed to improve the efficiency of nanoparticle synthesis by LRL. It was shown that the efficiency of nanoparticle synthesis by LRL could be improved by adding radical scavengers such as IPA to the precursor solution. This suggests that it is possible to synthesize nanoparticles in higher concentration solutions. In addition, it was confirmed that the addition of radical scavengers had no negative effect on the formation of solid–solution alloy nanoparticles, which is one of the characteristics of nanoparticle synthesis by LRL. It was also found that the addition of radical scavengers not only improves the synthesis efficiency, but also makes it possible to synthesize base metal nanoparticles, which have been difficult to synthesize in LRL due to re-dissolution of reduced atoms by oxidizing species. In the future, we will attempt to further improve the efficiency of nanoparticle synthesis by optimizing the volume of the solution, the concentration of precursor ions, and the type and concentration of additives. In this paper, we have demonstrated how to improve the efficiency of nanoparticle synthesis by LRL using a chemical approach. In addition, we will also investigate methods to improve the efficiency of nanoparticle synthesis by combining optical approaches such as laser scanning and multi-point irradiation.

## Experimental

### Materials

HAuCl_4_·4H_2_O (>99.9%, FUJIFILM Wako Pure Chemical Co.), H_2_PtCl_6_·6H_2_O (>98.5%, FUJIFILM Wako Pure Chemical Co.), Co(NO_3_)_2_·6H_2_O (>98.0 %, FUJIFILM Wako Pure Chemical Co.), HfCl_4_ (>99.5%, FUJIFILM Wako Pure Chemical Co.), Al(NO_3_)_3_·9H_2_O (>98.0%, FUJIFILM Wako Pure Chemical Co.), and YCl_3_·6H_2_O (>99.9%, FUJIFILM Wako Pure Chemical Co.) were used as precursor salts without any purification. 2-Propanol (IPA, >99.7%, FUJIFILM Wako Pure Chemical Co.), ethanol (>99.5%, FUJIFILM Wako Pure Chemical Co.), and glycerol (>99.5%, FUJIFILM Wako Pure Chemical Co.) were used as scavengers for the radicals generated by laser irradiation in the solution.

### Methods

Nanoparticles were synthesized by focusing and irradiating femtosecond laser pulses (Ti:sapphire laser, pulse width = 100 fs, pulse energy = 7 mJ, repetition rate = 1,000 Hz) on a precursor salt solution in a 3 mL fused silica cuvette or a 300 mL fused silica beaker. An aspherical lens with a focal length of 8 mm was used for laser irradiation on a fused silica cuvette, and an aspheric lens with a focal length of 8 mm was used for laser irradiation on a 3 mL fused silica cuvette. The UV–visible absorption spectrum of the solution during laser irradiation with a wavelength range from 300 to 800 nm was measured every 5 s using a fiber multi-channel spectrometer (FRAME-T, Ocean Optics Inc.). The nanoparticles synthesized by laser irradiation were observed using a transmission electron microscope (TEM, JEM-2100Plus, JEOL Ltd.), and the structure of alloy nanoparticles were evaluated using STEM-EDS analysis (ARM-200F, JEOL Ltd.).

## Data Availability

Data generated and analyzed during this study is available from the corresponding author upon reasonable request.
